# Hope in sight … Organisational challenges and mental health opportunities for refugee children in school contexts after arriving in an initial reception centre

**DOI:** 10.3389/fpsyg.2026.1721720

**Published:** 2026-08-19

**Authors:** Patrick Meurs, Dennis Schäfer, Deniz Özkan, Ina Kulić, Christine Bär, Julia Marburger

**Affiliations:** 1 Sigmund-Freud-Institut, Frankfurt, Germany; 2 Universitat Kassel, Kassel, Germany; 3 Katholieke Universiteit Leuven, Leuven, Belgium; 4 Odisee vzw Kenniscentrum Hoger Instituut voor Gezinswetenschappen, Brussels, Belgium; 5 Berufsförderungswerk, Frankfurt-am-Main, Germany

**Keywords:** hope, integration of refugee children in regular school, preparatory schools for refugee children, refugee children, resilience, school education, school education and mental health care, inclusion of refugee children in regular schools

## Abstract

**Introduction:**

We describe how school education for refugee children is often interrupted for long periods due to threats and insecurity prior to fleeing, discontinuity and dangers during the exile journey and the inability to immediately realize the universal right to school education for refugee children upon arrival.

**Methods:**

We provide an overview of policy-oriented literature on the organization of school education for refugee children. Between immediate integration into mainstream school education and completely segregated schooling, there is a broad spectrum of transitional, temporary, and parallel schooling options for refugee children. However, around half of refugee children worldwide do not have access to school education and those who finally start school, are confronted with language differences, stalled learning processes, psychological trauma, and vulnerability, either in themselves or in their parents. They also experience repeated school changes and can be victim of discrimination, and racism. We present our qualitative research method on the basis of interviews and drawing techniques for young children, investigating the meaning of school for refugee children, their parents, and teachers.

**Results:**

In the results of this qualitative research, we describe how school education during the initial arrival period can restore a sense of normality and provide hope for the future. Refugee parents, children, and careers link school attendance with hope and agency, as well as with developing a future perspective and revivifying of resilience.

**Discussion:**

We make it clear that the normalizing, hope-bringing and mental health-promoting aspects of any form of early preparatory schooling for refugee children are all too often being pushed into the background by the political conviction that direct integration in regular school classes is the only acceptable option, and by the educational science discussion about the most effective form of early schooling for these children (immediate entry into regular school classes or transitioning through temporary, preparatory school education in language and welcoming classes), a question that for several reasons has not yet been resolved and probably never can or will be.

*Illiteracy is like blindness*

(Soumaya, Afghan mother, 41 y., three children, in a reception centre since 3 months)

*I dream of creating something beautiful. With school training, I could improve myself and achieve some of my dreams*

(Rimas, Syrian adolescent, 14 y., in a reception centre with his family since 4 months)

## 1 Introduction

Mental health of refugee children is a well-documented area of research, as evidenced by the systematic review conducted by Dangmann et al. (2022). This review reveals a nuanced picture of heightened developmental risk alongside remarkable resilience—a combination also emphasized by Chen (2021). Refugee children are more likely to experience mental health issues in the post-flight phase, though the prevalence of these problems varies depending on the child's background, age, gender and the context in which they settle after exile (Frounfelker et al., 2020). Whether mental health issues develop or resilience flourishes depends on guidance, social support, opportunities available to the children, their parents, and their families, but also on personal characteristics and the daily structure and organization of their new living environment (Bronstein and Montgomery, 2011). In a first reception center, school education is an important aspect of refugee children's new living environment. Decision makers, developmental psychologists and educational scientists recognize the opportunity for refugee children to attend school and receive additional support for learning and psychosocial development, including school-based psychological preventive interventions: school attendance as a key factor in reducing developmental risk and promoting adaptive development among refugee minors from the arrival phase on (UNHCR, 2017; De Haene et al., 2018; Spaas et al., 2022).

The influx of refugees to Europe in 2015 and 2016—approximately half of whom were children and adolescents—catalyzed increased investment in the mental health of refugee minors, as well as in school education as a structured daily routine, a learning environment and an opportunity of delivering preventive interventions to refugee children. In this article, we focus specifically on school education as a source of hope and resilience for refugee children. However, from the outset of our research in this field, we realized that access to education is not guaranteed for refugee children, either in countries providing shelter within their own region or in the Western world. Fewer than half of refugee children worldwide attend school, and those who do often have to wait a considerable amount of time after arrival (UN, 2019). They do not always have equal access to the various levels and types of schooling in the country of arrival (UN Refugee Agency UNHCR, 2026). As a result, too many refugee children miss out on the resilience-building effects of school education.

Broadly speaking, the discussion about the optimal schooling of newly arrived refugee children in Europe revolves around direct integration into mainstream (regular) schools or a transitory phase in a parallel form of schooling that prepares them for integration into mainstream schools. What these visions have in common is the pursuit of inclusive school education for these children; the differences of opinion lie in the (political) decisions about when such preparatory schooling is to be established, in what form and for how long such a preparatory transitional form is to be offered, and whether it will continue to be offered in any way after inclusion of a refugee child in mainstream schools. Interviews with refugee children, their parents, teachers and staff members from initial reception centers reveal an additional perspective that is less often documented: school education can give refugee children hope and lead to resilience in the first months after they arrive. From the interviewees' point of view, these mental health aspects clearly outweigh the more theoretical and somewhat artificial, yet emotionally charged, discussion about the best school form for refugee children: direct inclusion in mainstream regular schools or only after a preparatory parallel school form. The experiences of refugee children in these preparatory classes can revitalize these children, shielding them from the effects of dehumanization and protecting them against parental distress and the effects of trauma. It offers them a sense of normality as well as a daily structure in their new living environment, opening up future perspectives of strength and hope.

The first part of this article describes how school education for refugee minors is conceptualized in policy documents, and how this differs from the reality of school education for refugee children. The second part illustrates how key stakeholders in schools (e.g., children, adolescents, parents, social workers and teachers) perceive schooling's contribution to refugee children's development after forced migration.

## 2 The gap between policy documents on school education for refugee children and the reality of preparatory schools for these children

According to Article 28 of the International Convention on the Rights of the Child (UNICEF, 1989), refugee children and young people up to the age of 18 have the right to a place in the school education system, regardless of their residence status. This right applies both to refugee children and young people who have only recently arrived in a country in which they intend to apply for asylum and to those who have been living there for several months or years and have already undergone significant parts of the asylum procedure. However, there is usually a waiting and transition period between refugee children being entitled to school education from the first day of their arrival and this right being implemented or realized. This waiting and transition period can vary from country to country. The point at which refugee children have the right to education (shortly after their arrival) and the point at which they become subject to compulsory schooling (shortly after being granted protection status and assigned a place of residence at a local level) are not the same. Additionally, some refugee children who do not receive preparatory or supplementary courses and enter mainstream education immediately often struggle to cope with the transition or catch up. During this waiting period before entering regular school, several countries offer various forms of schooling designed to support these children's transition to mainstream education. Consequently, there is a lot of variety in the organization of schooling that refugee children receive in the first period after arriving in their host country (Due et al., 2016).

Even though the registration of refugee families and the processing of their asylum applications takes some time after their arrival, there is consensus in many countries that the school education of refugee children should be realized in the meantime and within a few weeks (Crul et al., 2017; p. 62). Structural problems such as a shortage of teachers make this difficult, and organizing schooling in reception centers is not always easy either. At the same time, this education is important because it gives the children structure, enables friendships, helps to resolve stunted learning processes and, in addition to a sense of belonging, also offers a safe place to be a child. Without schooling in the arrival phase, there is a risk that the children's motivation to learn will be lost for a much longer period of time (Due and Riggs, 2013).

From a comparative international perspective, there are also differences in the extent of teaching immediately after arrival: sometimes a few hours a day, half a day or more; sometimes 2 or 3 days a week or every weekday; sometimes it is run by teachers with special training or by other professionals with short in-service training in the teaching of children with a refugee background, sometimes by professionals, sometimes by volunteers or a combination of both. Sometimes the first lessons take place in the reception centers, sometimes outside (even if the children are still in the reception facility). In most cases, schooling in the reception facility is preparatory schooling, but in some cases a department of a regular school has been set up in the reception center. Very often there is a clear desire to provide refugee children with school education, sometimes until the end of their schooling or compulsory education (at 18 years of age), sometimes even beyond (Crul et al., 2017). In some reception centers, the Ministry of Education is responsible for organizing this schooling in classes of the regular school that are attached to the reception center, while in other reception centers this initiative is supplemented and/or preceded by schooling organized by other ministries (e.g., the Social and Integration Ministry) or other agencies, sometimes (but not always) in cooperation with the Ministry of Education. The ideal or goal of integrating refugee children directly or as quickly as possible into mainstream education is often at odds with the reality of waiting periods before they can attend school and other practical obstacles such as a shortage of teaching staff in mainstream schools. Consequently, the specific provision of early school education for refugee children varies greatly across Europe and is subject to constant improvement or adjustment. Even in its current imperfect form, schooling in the initial period after arrival is extremely useful and important (Moro, 2012). How can the strengths of the various schooling programmes in the first months after arrival be preserved and subsequently utilized in such a way that refugee children can derive the greatest possible benefit for their future learning and developmental trajectory in regular schools?

To explore this question, we describe in the next paragraph the immediate precursors to the enrolment of refugee children in schools in reception centers: the focus on *child-friendly spaces* and other child-centered initiatives in refugee reception facilities in the German state of Hesse^1^. These child-friendly spaces promote development and learning. Despite these good initiatives and the focus on learning and child friendliness in the first reception centers in Hesse, this situation is not representative for the situation worldwide: less than half of refugee children worldwide have access to school education (Global Partnership Education (GPE), 2024). Another significant group of the worldwide child refugees' population has access to school education, but is taught in a completely separate and discriminatory system and has no opportunity to integrate into regular mainstream school education. The educational situation of refugee children is often not favorable in terms of chances of integrating refugee children into mainstream schools.

*Child-friendly spaces as a forerunner to development- and learning-promoting school education in initial reception centers for refugees*.

An important precursor to today's forms of education for refugee children in Hesse can be found in the years 2015–2016, when, at the height of the influx of refugees, attention was focused on safe *child-*friendly *spaces* in reception centers (Leuzinger-Bohleber and Andresen, 2017). As part of this approach, consideration was also given to educational opportunities that should be offered even before the children and their families were assigned to cities and municipalities, i.e., schooling in the reception centers. At the time of the 2015 refugee wave, there was not yet a systematic school educational provision in the reception centers that went beyond local voluntary work or local and individual initiatives.

The cognitive and linguistic support of children as a preparatory phase before integrating them into German regular schools only became the focus in a next phase, when policy makers recognized the need to structure the first dedicated voluntary initiatives at the local level, based on the question of how refugee children could best be integrated into the education system. Should refugee children be sent directly from the reception center to local mainstream schools after their arrival, or should this be done in stages, with a preparatory programme at the reception center until they can attend mainstream school once they have been assigned to a municipality? Or, should there be a learning provision at the reception center, followed by a transition to an integrative class^2^ at the local school outside the reception center and then into the regular school system? These many options reflect just as many different approaches that are already being practiced from a European perspective, even though there is currently a lack of empirical research on the effects of these different options on the later educational success and the general development of refugee children. Moreover, the question of the most effective school system may not even be the most central issue, since early school education for refugee children is primarily effective due to its mental health benefits (resilience, self-esteem, coming to accept a clearly structured daily activity after the chaotic survival during the flight, feelings of belonging and participating, hope for a better future, safety in a predictable environment).

*The limited number of refugee children who have access to school education worldwide*.

When refugees leave their homeland, many plans for the future are put on hold. The search for a safe place to stay is now the priority. Children who are part of such a refugee wave have often not attended school for a long time due to various threats in their country of origin. In addition, they are confronted with the fact that their school education has been interrupted during their flight and that their further education and learning in the country where they arrive and apply for asylum may be hampered by increased barriers (poor language skills and lack of prior knowledge in certain subjects on the side of the children, lack of knowledge of the school system among the parents, lack of resources on the side of teaching staff). The schooling of refugee children also requires special investment on the part of the host country, but even when schooling is offered, there is a risk that children will end up in overcrowded classes and financially less supported forms of education and that they will be confronted with their own inner limitations, such as language differences, social exclusion or the consequences of trauma (UNHCR, 2020). More than half (!) of all refugee children worldwide do not attend school, even though it has been repeatedly shown that schooling is crucial for their later participation and integration into society (UNHCR, 2023).

Refugee girls are particularly disadvantaged for several reasons (UNHCR, 2024): their access to education is significantly lower than that of refugee boys. Education for refugee children has only been higher on the agenda internationally and in individual countries very recently, since 2017—just a few years after the so called “summer of exile” in 2015 in Europe (UNHCR, 2017).

The above-mentioned UNHCR reports state that, in an international comparison, 38% of refugee children between the ages of three and six currently receive some form of education, while the figure for primary school age refugee children is 65%. After that, the proportion drops rapidly to around 40% in the mid-teenage years (15 and 16-year-olds) and only 6% among those over 18. This decline is attributed to the lack of an inclusive school policy in many refugee-hosting countries, capacity problems in schools, the increasing cost of schooling for parents as children get older, administrative hurdles and, in the case of girls, certain gender-specific stereotypical expectations and prohibitions.

Despite these figures, which indicate considerable difficulties in the educational participation of refugee children, initial research findings show that the integration of refugees into the school system is beneficial both for the refugees themselves and for the host society, e.g., by promoting social cohesion and tolerance, increasing the self-esteem of refugee children and improving their future career opportunities (Kirdar et al., 2021). Furthermore, the gap between what refugee children and children without a refugee background benefit from school education becomes smaller the earlier after arrival school education for refugee children begins and the younger the children are (Figlio and Özek, 2020).

In countries such as Sweden and Finland, where refugee children are integrated into the regular primary school system very quickly, the success rates also appear to be significantly better than in countries where the school integration of refugee children begins later and often in preparatory or transitional language classes. At first glance, this is an important argument in favor of integrating children into regular mainstream schools as quickly as possible (Agirdag, 2020; Höckel and Schilling, 2022). However, as we will see, the research basis for this claim is still far too uncertain or even almost inexistent, and the direct translation of this finding into recommendations that would then apply without question throughout Europe or the world and lead to the same result everywhere is neither given nor possible in practice.

Overall, more and more refugee children and youngsters are coming into our society who have experienced trauma, adversity and disadvantage early in their development (*children with early adversities, children at risk)*. If we do not help them early on to find their way into our schools, a significant proportion of them will enter society without an education, with all the consequences that this entails. If we do not provide them with transitional spaces to prepare them for this path to school, or if we do not put in place accompanying measures after their transition into the mainstream regular school system, there is a risk that too many of them will get stuck in school, regardless of the success stories of some very gifted refugee children. There is also a risk that too many teachers and too many schools will be overwhelmed by the special needs of refugee children.

*Inclusion of refugee children into regular schools, either directly or through preparatory transitional forms of schooling?*

Despite many refugee children struggling to access mainstream education and a lot of schools being ill-equipped to support them, the position of the United Nations and the UNHCR is clear. They recommend giving refugee children priority access to mainstream education. The discrepancy between these recommendations and the practical problems faced by schools confronted with large numbers of refugee children is striking and requires an explanation. It is a discrepancy that can only be understood in the context of certain global trends against which the UN wishes to act, such as the segregation, discrimination and racism experienced by refugee children in schools around the world or the impossibility for refugee children to gain even the slightest access to the mainstream school system. These trends will therefore be discussed first.

The older refugee children are before they can start school, the more difficult it is for them to enter an age-appropriate form of education. More than half of refugee children do not attend an age-appropriate school by the age of 15, while this proportion is significantly lower for children without a refugee background. While 91% of primary school children aged 6 to 10 worldwide attend school, the figure for refugee children is only 65%; for young people aged 11 to 18, the difference is even more pronounced: 84% worldwide, but only around 40% for refugee youngsters of 16 years, only 25% at 17 and 18 years Grandi (2020). A 2019 UN report speaks of 7.1 million refugee children, 3.7 million of whom do not attend school (UN, 2019). In this report the United Nations, High Commissioner for Refugees, Grandi (2020), advocates integrating refugee children into the national education system as far and as soon as possible, rather than keeping them in completely segregated systems, as is the case, for example, with Syrian children, who are often never admitted to the regular school system in Turkey.

In contrast to such completely segregated andnot seldom discriminatory systems, in which the number of school dropouts is high, Grandi (2020) emphasizes the importance of inclusion in the regular education system in order to prevent them from becoming trapped in parallel worlds as early as kindergarten or primary school, from which integration into society is more difficult. However, the need to counteract the aspect of exclusion, discrimination and segregation in parallel schooling forms—as is obvious in the UN Report - carries the risk that any form of parallel schooling, including preparatory, transitional and supplementary education, will be rejected as inappropriate or even unacceptable. As a result, the positive and perhaps even necessary aspects of such schooling disappear from view and are no longer considered or examined. Our plea to reconcile the best of both forms of education should also be understood from this perspective. Where integrative education is already offered on a larger scale, as in several EU countries, there is also a growing awareness that such integration brings new challenges and may only be possible if these children are prepared for integration into mainstream regular schools, either through a transitional parallel form of education or through additional support for the mainstream school system that refugee children enter, as is the case in Norway, Sweden and Finland, for example (OECD, 2019). The example of these Scandinavian countries, where the direct integration of refugee children into mainstream education has led to positive results, is misleading, because in other EU countries mainstream education does not always receive the same level of financial support to cater for this group of refugee children.

The European Commission's Action Plan *on Integration and Inclusion* (for the period 2021–2028), adopted in 2020, places the responsibility for implementation primarily with the European Member States. Among the strategies and measures proposed in the Action Plan, the emphasis on building *learning networks* is particularly noteworthy: integrating large groups of children with very different backgrounds and school experiences into the mainstream education system requires exchange and the opportunity to learn from each other, in a broad learning network of teachers and other school staff at national and international level. The EU document focuses on early language learning, recognition of the mother tongue, recognition of schooling prior to flight, respect for diversity, building teaching capacities, and networking on this topic (e.g., in the form of roundtable discussions European Commission European Education Area (2020), refresher courses, case studies, supervision, reflective spaces, opportunities for teachers to exchange adapted learning materials, etc.).

In a World Bank Report, Bentaouett-Kattan and Oviedo (2023) describe that many of these children live in a prolonged period of crisis and uncertainty. Some of them have been displaced throughout their entire school years, so that—once school attendance becomes possible—integration into a school system is not a given, but nevertheless extremely important. In addition to a clear national inclusion policy and targeted educational support for refugee pupils and inclusive schools, there is a need to exchange best practices in this area. The authors of the World Bank Report discuss the importance of a transition from temporary preparatory forms of education to regular mainstream schools. This transition from non-formal and mostly parallel forms of education to a state-administered and inclusive education system is of great importance, even though the authors point out that actual research into the modalities of this transition is still in its infancy.

With this terminology, we have referred to the two main models of schooling for refugee children in Europe and thus also in Germany or in Hesse: the parallel and the integrative models (Massumi et al., 2023). Unlike with the parallel-segregational form of school education for Syrian children in Turkey, the parallel-inclusion oriented European and German models are much more focused on introducing these children to regular schooling. In Germany, such a parallel model means creating a temporary and safe transition space, offering German language and mathematics courses and protecting against uncertainties (e.g., discrimination, exclusion, bullying). In addition, it represents a temporary preparation for the transition to mainstream school. These parallel models are often contrasted with integrative models, in which integration into mainstream school is the focus from the outset, although this is often accompanied by parallel additional language support alongside regular classes, which refugee children attend either fully or partially, as is the case in Sweden, for example.

The duration of parallel preparatory schooling should not exceed 12–24 months. According to (Höckel and Schilling 2022), two advocates for direct inclusion in regular schools, less than 20% of children in parallel primary school classes for refugee children transfer to general secondary schools at the age of 11 or 12 (the level of secondary school with the broadest range of possibilities). This is significantly lower than the proportion of all children in Germany who attend this kind of secondary schools, which is between 50 and 55%. At age 10, the school performance for German language of children from parallel primary school classes is 0.20 SD below that of refugee children who transfer directly to mainstream primary schools before the age of 10. Surprisingly, these children in parallel classes (with mainly German language courses) perform worse in German than refugee children who enter mainstream schools directly without 1 or 2 years of intensive language instruction in parallel classes. The researchers suspect that this result is due to the lack of informal German language exchange with peers during and after school hours in a parallel class consisting only of refugee children (Schilling, 2022).

Based on these research results, one might ask why direct entry into mainstream schools is not chosen more often. However, this may also be a purely research-based conclusion, supported by only very few empirical research. The researchers mentioned above (Höckel and Schilling), showing the better cognitive and learning results in case of direct inclusion in mainstream school education, admit that they did not examine the psychosocial or mental health benefits of temporary parallel support in their study. Nor do they describe how parallel provision can prepare or sustainably support the regular school career of refugee children. Last but not least, parallel forms of education may be slightly less efficient because they do not have the resources to provide adequate care for so many children, because they employ less trained teachers, and because this system, which has worked with smaller groups of refugee children, may not have been prepared for the large influx of these children since 2015. In this sense, we should not throw the baby out with the bathwater and ask ourselves, how we can best organize this preparatory, bridging and/or additional schooling for refugee children?

School attendance usually only begins once families and their children have been assigned to local authorities. Höckel and Schilling (2022) found that refugee children in Germany start school an average of seven and a half months after arriving. They are more likely to be below their expected level; for example, 40% of 15-year-olds with a refugee background are in the Levels 6–8 (for 12–14 year old youngsters), compared to 6% of children without a refugee background. Therefore, the reality that a direct transition to mainstream school also has disadvantages and takes time to become possible, is difficult to ignore.

There is another reason to conclude that direct access to mainstream education is not yet feasible in many cases. This concerns a number of refugee children in Europe who never attended school before their arrival, such as some children from nomadic families in the war zones of the Middle East, as well as children who have become war orphans in the (Syrian) civil war and have not attended school for a very long time when they are brought to Europe for various reasons. Other children are very traumatized by dehumanization. All these sub-groups of refugee children still need to be prepared for a daily routine and, some of them, introduced into trustworthy relationships. In all these cases, regular school is not yet an option and even preparatory classes in the reception center are too ambitious. They require preparatory school education to develop the necessary structure and social abilities to function adequately in a reception class and begin learning. In these cases, mainstream education is preceded not only by preparatory school education, but also, in very specific cases, by an initial building up of a relationship between an adult and a child, a teacher and child^3^

While the preparatory phase of schooling cannot be replaced immediately by regular schooling for several reasons, the question remains as to how this preparatory and parallel schooling programmes can be organized in the most meaningful way. This will involve avoiding the pitfalls of parallel education systems, such as a lack of contact with local children, a high staff and student turnover, a lack of qualified teachers and of networks between teaching staff, or an educational content that often remains limited to language teaching.

## 3 Education for refugee children as a source of hope, agency, future perspective and adaptive development

For parents with a refugee background, their children's school education is a very high priority. To understand the perspectives of refugee children, their parents and teachers, the following sections present the results of a qualitative research component of a broader research project on preparing for integration processes for children, young people and adults from the moment they arrive at a reception center^4^.

*Qualitative content analysis of interviews with refugee children and their parents/carers about school education*.

When the interview material from refugee children and their parents was first examined, an interpretation group identified several themes that stood out immediately because they were mentioned by more than one respondent. Concerning school education, the topic of hope was particularly prominent, as a more detailed content analysis soon confirmed.

This content analysis was carried out in accordance with the approach of Mayring (1994), who defines this method as “qualitatively oriented, category-guided text analysis.” The term “category-guided” means that a category system must be developed by forming and compiling categories from the data, an approach that is systematic and strictly rule-based. Following this approach, inductive category formation is closely tied to the interview material in the sense that categories are derived directly from the empirical data in an active construction process. Accordingly, this approach aims to reduce the material to its essential content, abstracting manageable statements that still reflect the source material. These categories are understood as statements which contain the core messages of the material in condensed form (Kuckartz and Rädiker, 2022).

Initially, the individual responses were coded to establish a category system using inductive content analysis. To this end, each response was coded^5^ and provided with a paraphrase of the statement. The premise for this step is that the paraphrase must be very close to the original text and must not contain any interpretative attributions. This process was repeated for the entire data set.

The next step was to generalize and summarize the content of the paraphrases through collaborative discussions within the research group, and then to abstract and categorize them in an initial reduction. For example, the paraphrases “to study” and “to be able to continue one's education” were summarized as “education/study.” Once the data had been categorized in this way, the final step was to hold discussions within the research group to derive second-order reductions based on the first-order reductions that had been developed previously. For example, the overarching category “professional future” was formed based on the first reductions “education/study” and “work.”

While we will not present the full results of this content analysis, we will note that the category “hope” featured frequently in the responses. This category is linked to “agency,” or the ability to look ahead, keep moving forward and stay motivated. Rather than focusing on psychological vulnerability and victimhood, interview fragments surrounding the theme of schooling emphasize hope and agency. For children and parents alike, schooling is often associated with future prospects for the family, as well as a better chance of being recognized as asylum seekers with protected status. For parents in particular, hope is most frequently associated with their children's ability to attend school in Europe.

*School education for children as a sign of hope and restoration of continuity and normality*.

In the initial reception centers in Hesse, various professional groups are involved in the schooling of refugee children: teachers with teacher training, but also other professionals such as social workers, educational assistants or volunteers with or without additional training in teaching in a school context. Each of these groups includes some individuals with a refugee background. By teaching in schools for newly arrived refugees, these teaching persons with a refugee background wish to give something back to the country that offered them shelter and opportunities, years ago. A recurring theme in interviews with these teaching assistants—whether they have a refugee background or not - is the hope that education in the initial phase after arrival will promote the later learning process of the refugee minors, primarily by laying a foundation in language and mathematics, but also by supporting the development of these children in general.

For the sample of refugee children we studied, attending school is associated with social contacts, belonging to a group of friends, learning a new language and other forms of expression (e.g., creative design, drawing and music), and having a place of refuge where they can temporarily leave certain other worries behind.

Rahim^6^ (Iraq, 9 years old): “School is fun.” Interviewer: “Okay, tell me what is nice about school?” R: “Because we learn there and when we argue, the teachers intervene and resolve it. There are also fun games and there is a forest where we can play and run around a lot. And we learn every day. Every day I learn a few new words.”

It is also very important for these children to experience that their teachers want the best for them and want them to progress in their development. Some children say that they cannot imagine how long the day would be if they could not go to school in the reception center.

Interviewer: “What does school mean to you?” Fatima (Palestine, 16 years old): “It's like the only big hope I have left, it's also the only normal thing, like my old life was, because what's here at the center now is so different from my life in Gaza. (...) I can go outside, leave the facility, and be at the village school there, every day, about half the day. School is quieter than the center, there are fewer people with problems. At school, I don't have to be in the noise of the center and hear the children screaming all the time, because then I think too much about what I saw in Gaza... At school it's quiet and I can think on what the teachers tell us and I can reflect about my life.” I: “Think about what?” F: “I just think about what I want to do next, continue studying... I hope I can stay here at this school for a while longer, but you never know.”

These children are generally aware that the opportunities offered to them by school are fragile.

“I have high hopes here in Germany: finishing school, a career, a girlfriend—the things you need to build a better future. (…) But when you're a refugee, you never really know what tomorrow will bring.” (Afghan young man, 16 y).

It is also striking that, for most of the refugee children we interviewed, school, sport, health, space to live and time for reflection, social contacts, and the opportunity to stay active and make plans are closely linked, as if school helps to maintain these healthy and social aspirations, which are important for recovering a healthy mental state.

Parents also recognize how important school is for their children and how starkly this contrasts with their own experiences when they did not have these opportunities.

“Because I myself did not have the opportunity to go to school and be strong in life, I want my daughter to continue going to school and get a good education. (Somali father, 28 years old, one child aged 5)

The contacts in the school of the initial reception center make it very clear to most parents that the school system in the country to which they have fled works differently from what they themselves are familiar with. Against this backdrop, it is understandable that parents fear their children will struggle at school and that, in this case, their asylum application will be rejected, extinguishing the hope that starting school had offered them.

The Afghan mother quoted at the beginning of this article explained that as a refugee, it is only through school education that one can be empowered and participate in society with all one's intellectual abilities. She spoke of her pain as an Afghan woman who was unable to receive an education and described the consequences (illiteracy) as a form of blindness. The opportunity for schooling that her children had in the initial reception center was a great ray of hope for this mother. She put it as follows:

“Illiteracy is like being blind. By sending my children to school, there is hope of no longer being blind, of seeing new perspectives and ways into a safer future.” (Soumaya, 41 years old, Afghanistan, three children).

Some parents also make it clear that, despite all their grief over experiences of loss and the effects of possible trauma, they and their children draw resilience and strength from the opportunities offered by school education. But, who can help these parents support their children in overcoming the difficulties on a path that the parents themselves were sometimes unable to take, who can help them deal with the uncertainty of whether they will ever really count in this society, even if they are successful at school? These doubts and uncertainties do not change the fact that school education and (vocational) training are of enormous value to these parents and children: it means having the ability to see, to have a voice, to look ahead, to have a perspective and to work and participate within the society that they hope to become members of.

*It takes a whole village to teach a refugee child*.

As part of our research project, we also talked to refugee children in the first years of primary school, about how they experience school and what their expectations of school are. First, we helped them paint a picture of a school building and use this picture as a basis for a conversation about their current or past school experiences and their future wishes for school. If we are unable to connect with the child through the drawing, we continued to build the researcher-child relationship with the help of puzzles and games. Building a relationship of trust is very important in order to be able to talk about school at all.

The parents associate learning successes of the children with the good accessibility and visibility of the teacher in the initial reception center. For parents, quick access to school education in the initial reception center is associated with hope, but often also with very high expectations of their children: hope for the whole family, the idea that success at school means salvation for the whole family, with images of very ambitious professions for their children (doctor, judge, engineer, and architect, etc.). For example, a social worker at a reception center in Hesse recounts:

“I knew a 14-year-old Syrian girl who was extremely talented at drawing and art. However, her parents saw art as a “useless pursuit,” literally “a breadless activity,” something you couldn't earn a living from, not even pay your food from. They told her that she had to become an engineer or a doctor. Not even a psychologist, because that profession was considered worthless too. It took a long time, but I was able to talk to the parents often and they finally agreed to send their child to art school. It was good for her, as you could see from the hope she now had, and her parents' horizons had also broadened. Our conversations about the choice of school turned out to be important for the mental wellbeing of all concerned, because these conversations yielded results.”

Yet, attending school is not easy for some refugee children in an initial reception center, regardless of whether the school is located inside or outside the reception center. Sometimes these children are caught up in loyalty to a parent who is having a difficult time due to trauma or traumatic bereavement processes. Especially for girls who take on a lot of responsibility and look after the whole family when a parent is unable to do so, the involvement of a support person is very important in helping these children to attend school. Teachers feel that these children cannot find the inner peace to absorb what the teacher is offering them. In such cases, it is particularly important for teachers to be able to rely on other colleagues, volunteers and mentors who can support these children in their school attendance and learning process.

“Among our colleagues [...] we are all paired up with one of the children in the center. Several colleagues have one or one and a half hours per week to discuss homework with this child. On the other hand, I also lead the volunteer programme, which includes a number of people who come to our pupils' living places on some evenings during the week to help them with their homework or pick them up from their families in the morning, to go to school. So all of this is happening, but even then we notice that for some children it is not so obvious that they are fully committed to attending school. We find that sometimes there are children who don't come to school for weeks. When you ask them about what is going on, they say, “No, no, everything's fine,” until we hear from the teachers that they haven't been there for 2 or 3 weeks. So not everything is running smoothly, and I think that also has to do with the role of the parents. Parents who oblige their children, i.e., in the sense of “now you go downstairs, you sit with your school bag ready until the supervisor comes,” this is not a matter of course for some parents who are themselves mentally stressed or traumatized or who are overburdened by raising their children alone.” (Social worker and teacher at an initial reception center in Hesse).

Some refugee parents also report that, in addition to the hopes they place in their children's school careers, they are very concerned that their children will encounter everyday obstacles that will cause them to fail in their learning process. The regular transfers from one reception center to another or to a new home also create a discontinuity that adds to other experiences of loss and is therefore sometimes unbearable for these children and their parents.

“I moved every 3 months... a really difficult situation, also for my children. In 2 years, they changed schools five times. Really very difficult. [...] New place, new bed, new friends, new teacher, new school. [...] I am also tired and sad that my children have to change schools so often. And we see our child crying and hear: “Mummy, I don't want to change. I don't want to leave my friend... When I hear that from my child, I lose all hope for my child's future.” (Sonia, 34 y., single mother of two primary school-aged children, from Bosnia).

This Bosnian mother expresses what the United Nations High Commissioner for Refugees, Filippo Grandi, has been emphasizing for years as a central pillar of a humane reception of refugees: school education for refugees gives hope and perspective and embodies the host countries' investment in the safety of these vulnerable people after their flight.

“School is often one of the first things refugee families look for after their displacement. After the trauma of uprooting, they want to regain a sense of normality and dignity and invest heavily in their children's future. Many children and young people were displaced several times before crossing a border and becoming refugees. For these children, school is the first place where they can find routine, safety, friendship and even peace again. School education plays a key role in protecting children and young people and helping families and children to focus on rebuilding their lives with hope.” (UNHCR Filippo Grandi (2020), no page number www.educationcannotwait.org/ September 2020; Grandi (2022)).

## 4 Conclusion

*Recognizing the invaluable preparatory school education provided in initial reception centers as a source of experiential knowledge to support subsequent mainstream education and as a context of hope for learning and developmental processes of refugee children*.

Integrating refugee children and young people into mainstream schools as quickly as possible is a demand that often appears in research literature and political documents. At the same time, there is a growing recognition that this is not immediately achievable, even though we consider it a highly desirable goal that from an ethical point of view should be pursued as a matter of priority and as a sign of an equal chances policy. Because a direct entry into regular schools is mostly unrealistic for several reasons, we must preserve the wealth of experience gained with preparatory forms of schooling and transfer these experiences to the next step for these refugee children in regular schools. These preparatory transitional forms are very useful if they are limited in time, offered or supervised by well-trained teachers, form a bridge to mainstream schools and can continue to be offered even after the transition of the child to mainstream schools. This requires continued and possibly increased investment from various political bodies involved in the school career of refugee children, an investment that is necessary to increase the success rate of refugee children in our school system and thus maintain the hope that our schooling system inspires in refugee parents and children.

These children, youngsters and parents, who have repeatedly been confronted with interrupted educational and future prospects, are trying to make sense of their life experiences and redesign their future plans. They do this from the moment they arrive at a reception center and do not yet have a clear idea of their prospects for staying. At this point, the new opportunities for school education for children and young people have the potential to create and strengthen hope and new prospects (Thoma and Langer, 2022). School programmes and the transitions between school systems are particularly important in this context, which is precisely why diverse forms of early schooling may not be a bad thing: these diverse forms are also an expression of the desire of many providers to shape these hopeful prospects for refugees.

Thanks to the commitment of the many counselors, teachers, educators, social workers and volunteers in the reception centers and the humanity they embody, this kind of preparatory school education, early after arriving, represents hope. It makes ambition and future inclusion conceivable, essential elements for restoring mental health in refugee children and their parents and for finding a way to deal with cumulative loss, trauma and unfinished mourning processes (see also: UN, 2019; UNESCO, 2026).

At the same time, this hope may be temporary (Hettich and Meurs, 2020), due to the many changes of residence in the first months after arrival, especially when these people, after being transferred from the reception center, no longer feel as secure and supported within broader society as they did before in the first reception center, but also due to the unrealistic expectations of parents (who only consider the most highly regarded professions to be good enough for their children), the tendency of children to put themselves aside in order to care for their traumatized parents, or the lack of a professional exchange between the initial school education in the reception centers and the subsequent school education in mainstream regular schools (too little exchange of experiences, information, teaching materials, etc., as a result of which opportunities for teachers or schools to learn from each other about teaching refugee children remain unexploited or do not arise at all). The current shortage of teachers in schools, the difficulty of the decline of the number of teacher training candidates, and the early departure of demotivated teachers due to budget cuts in this field threaten to undermine the quality of school education in many European countries. Political decisions to lower budget for school education in general could have the greatest impact on the most vulnerable children, such as refugee children.

Consequently, refugee-oriented school education must remain an ongoing concern in societies that want to remain open to exiled people. This is not a self-evident political choice at a time when various countries are influenced more or less by an atmosphere of canceling diversity, an extremist and intolerant atmosphere which also puts pressure on the hope and mental health of these refugees.

The reality of preparatory school education, in which many people are deeply committed to helping vulnerable refugee children, lies between the desire to offer refugees equal opportunities by direct access to mainstream education, and the misguided, inhumane and discriminatory denial of any such opportunity for integration in mainstream school. Preparatory and supportive forms of school education have often arisen from the needs on the working floor of schooling, but they are insufficiently researched in the somewhat pompous discussion about whether or not to grant direct access to mainstream education. To respond to the multifaceted real needs of refugee children, we must pay attention to the experiences of these types of preparatory education and the lessons learnt from them. We must speak about the richness of these forms of school education in the context of research, in the negotiations with policymakers, in the training of teachers, and among mental healthcare workers, who not seldom come into contact with these refugee children at school and see the school not only as a context of education but also as a primary mental health care setting to reach this target group (Murray et al., 2010; Spaas et al., 2023).

Our qualitative research reveals that adopting a hope-based approach within collaborative networks involving schools and mental health services is crucial for preventing long-term psychological problems in refugee children, as well as for promoting learning and developmental processes. Hope motivates displaced people and is essential for resilience, providing a tangible alternative to experiences of loss, homelessness, trauma, and other hardships endured before, during and after flight. This finding is consistent with recent research into refugees, which suggests that hope is a protective factor that promotes positive psychological functioning and resilience (Chen, 2021). Although much research on refugees has focused on their psychological distress and the risk of psychopathology, the role of hope-based interventions in a school context and in first reception centers warrants further investigation (Dimoski et al., 2025; Bateson et al., 2026).

## Data Availability

The datasets presented in this article are not readily available because the data are only available for the scientists in our own research project. Requests to access the datasets should be directed to patrick.meurs@uni-kassel.de, patrick.meurs@kuleuven.be.

## Footnotes

1 Hesse is a state in the centre of Germany, with cities such as Frankfurt, Darmstadt, Gießen, Kassel, Fulda, and the capital of this state, Wiesbaden.

2 In Hesse: InteA-klassen—Integration mit Anschluss und Abschluss—Integrationclasses with Connection and Completion; in Belgium: OKAN Onthaalklassen Anderstalige Nieuwkomers—Reception classes for foreign language newcomers. In the German words ‘Anschluss und Abschluss' are implicated: re-connecting to a school system and finishing this preparatory school system in order to enter a regular school system, and also having completed a certain level of language acquisition.

3 A good example of this preparation for a preparatory reception class is ‘La petite école' (‘Te small school') in Anderlecht Brussels. This form of schooling is demonstrated in the reportage ‘Les éclaireuses', which showcases an accessible, small-scale school that admits refugee children unable to cope in regular or preparatory first school education. See: https://www.vrt.be/vrtmax/a-z/eclaireuses/.

4 This study is part of the wider research project ‘Transitional Spaces for Further Arriving' (2022–2026). On the one hand, this project comprises a quantitative research component investigating the mental health issues experienced by refugees in the arrival phase, on the other hand a qualitative research component with open-ended interview questions about the life experiences of refugees in initial reception centres. The latter includes questions for children and parents regarding school attendance during this period. The project is funded by the Hessian Ministry for Social Affairs and Integration.

5 Using MAXQDA, a software programme for mixed-methods research and qualitative data analysis, in which, for example, interview data can be transcribed, analysed, coded and then presented according to main and sub-categories of content.

6 The names of the interviewees have been anonymised.

## References

[B1] Agirdag O. (2020). Onderwijs in een gekleurde Samenleving [School education in a diverse society]. Berchem: EPO. Dutch.

[B2] Bateson M. Casley M. Cartmel J. Mc Gregor G. (2026). Interdisciplinary collaboration in school mental health: a scoping review. J. Interprof. Care 40, 551–564. doi: 10.1080/13561820.2026.2633621 41733193

[B3] Bentaouett-Kattan R. Oviedo M.-E. (2023). Rising to the challenge: protecting refugee children's education amid fragility, conflict and violence. New York, NY: World Bank. Available online at: https://blogs.worldbank.org/en/education/rising-challenge-protecting-refugee-childrens-education-amid-fragility-conflict-and-violence (Accessed May 25, 2024).

[B4] Bronstein I. Montgomery P. (2011). Psychological distress in refugee children: a systematic review. Clin. Child Fam. Psychol. Rev. 14, 44–56. doi: 10.1007/s10567-010-0081-0 21181268

[B5] Chen A. (2021). Risk and resilience of refugee children surviving war and displacement. (doctoral dissertation). Harvard University Graduate School of Arts and Sciences, Cambridge, MA, United States. Available online at: https://dash.harvard.edu/entities/publication/63807e00-1042-4500-8be7-6cfded84cb0 (Accessed on March 08, 2026).

[B6] Crul M. Keskiner E. Schneider J. Lelie F. Ghaeminia S. (2017). “No lost generation? Education for refugee children. A comparison between Sweden, Germany, The Netherlands and Turkey,” in The Integration of Migrants and Refugees, eds. R. Baubock, R. and M. Tripkovic (Fiesole: European University Institute + EUI Forum on Migration, Citizenship and Demography), 62–80.

[B7] Dangmann C. Dybdahl R. Solberg Ø. (2022). Mental health in refugee children. Curr. Opin. Psychol. 48:101460. doi: 10.1016/j.copsyc.2022.101460 36130437

[B8] De Haene L. Neumann L. E. Pataki G. (2018). Refugees in Europe: educational policies and practices as spaces of hospitality? Eur. Educ. Res. J. 17, 211–218. doi: 10.1177/1474904118762825

[B9] Dimoski J. Vukčević Marković M. Stojadinović I. Milić A. Adjuković D. (2025). Conceptualization of hopeamongadolescents and youth from refugee backgrounds in a transit context. J. Child Youth Care 171:108171. doi: 10.1016/j.childyouth.2025.108171

[B10] Due C. Riggs D. (2013). Care for children with migrant or refugee background in the school context. Adelaide. Available online at: https://damienriggs.com/blog/wp-content/uploads/2013/09/Experiences-of-school-belonging-for-young-children-with-refugee-background_author-accepted-version.pdf (Accessed March 20, 2024).

[B11] Due C. Riggs D. Augoustinos M. (2016). Experiences of school belonging for young children with refugee background. Educ. Dev. Psychol. 33, 33–53. doi: 10.1017/edp.2016.9

[B12] European Commission and European Education Area (2020). Refugee and migrant integration into education and training. Brussels: European Commission. Available online at: https://education.ec.europe.eu/focus-topics/improving-quality/inclusion-education/migrants-and-refugees (Accessed March 20, 2024).

[B13] Figlio D. Özek U. (2020). An extra year to learn English? Early grade retention and the human capital development of English learners. J. Public Econ. 186c, 5–32. doi: 10.1016/j.jpubeco.2020.104184

[B14] Frounfelker R. L. Miconi D. Farrar J. Brooks M. A. Rousseau C. Betancour T. S. (2020). Mental health of refugee children and youth: epidemiology, Interventions and future Directions. Annu. Rev. Public Health 41, 159–176. doi: 10.1146/annurev-publhealth-040119-094230 31910713 PMC9307067

[B15] Global Partnership Education (GPE) (2024). Education for refugees. Available online at: https://www.globalpartnership.org (Accessed October 9, 2025).

[B16] Grandi F. (2020). Education cannot wait. New York, NY/Geneva: UNHCR. Available online at: https://educationcannotwait.org/news-stories/featured-content/education-cannot-wait-interviews-the-united-nations-high-commissioner-refugees (Sept 2026) (Accessed March 28, 2024).

[B17] Grandi F. (2022). Rifugi e ritorni. Milan: Mandadori Store. (E-book)

[B18] Hettich N. Meurs P. (2020). Complex dynamics in psychosocial work with unaccompanied minor refugees with uncertain future prospect: a case study. Int. J. Appl. Psychoanal. Stud. 18, 41–57. doi: 10.1002/aps.1676

[B19] Höckel L. S. Schilling P. (2022). Starting off on the right foot: language learning classes and the educational success of immigrant children. Essen: RWI Leibniz Institute for Economic Research. ISBN 978-3-96973-148-2 (Ruhr Economic Papers, No. 983. (Accessed August 2, 2024).

[B20] Kirdar M. Koc I. Dayioglu M. (2021). School integration of refugee children: evidence for the largest refugee group in any country. Istanbul: Koç University Press and Tusiad Economic Research Forum. (Tusiad Working Paper No. 2116). doi: 10.2139/ssrn.3921506

[B21] Kuckartz U. Rädiker S. (2022). Qualitative Inhaltsanalyse. Bazel: Beltz Juventa.

[B22] Leuzinger-Bohleber M. Andresen S. (2017). STEP-BY-STEP. Final Report on the Step-by-Step Pilot Project for the Care of Vulnerable Traumatised Refugees in Hessian Initial Reception Facilities. Wiesbaden: Hessian Ministry of Social Affairs and Integration.

[B23] Massumi M. Brandl C. Korntheuer A. (2023). “Models of school integration for refugee children and youth in Germany. Identifying gaps in the current state of knowledge,” in The Research Handbook on Migration and Education, eds. N. Bunar, H. Pinson, and D. Device (Cheltenham Glos: Edward Elgar Publishing), 234–251. doi: 10.4337/9781839106361.00010

[B24] Mayring P. (1994). “Qualitative inhalsanalyse,” in Texte Verstehen: Konzepte, Methoden, Werkzeuge, eds. A. Boehmer, A. Mengel, and T. Muhr (Konstanz: Univ. Verlag Konstanz), 159–175.

[B25] Moro M. R. (2012). Les Enfants de L'immigration: Une Chance Pour Nos Écoles. Paris: Bayard.

[B26] Murray K. E. Davidson G. R. Schweitzer R. D. (2010). Review of refugee mental health interventions following resettlement: best practices and recommendations. Am. J. Orthopsychiatr. 80, 576–585. doi: 10.1111/j.1939-0025.2010.01062.x PMC3727171 20950298

[B27] OECD (2019). Indicators of inclusion in education: a framework for analysis. Paris: Organisation for Economic Co-operation and Development. Available online at: https://www.oecd.org/en/publications/working-together-for-local-integration-of-migrants-and-refugees-in-paris_9789264305861-en.html (Accessed March 28, 2024).

[B28] Schilling P. (2022). Integrating refugee children into schools. Available online at: https://blogs.worldbank.org/en/impactevaluations/integrating-refugee-children-schools-guest-post-pia-schilling (Accessed July 9, 2026).

[B29] Spaas C. Said-Metwaly S. Skovdal M. Primdahl N. L. Jervelund S. S. Hilden P. K. L. . (2023). School-based psychosocial interventions' effectiveness in strengthening refugee and migrant adolescents' mental health, resilience, and social relations: a four-country cluster randomized study. Psychosoc. Interv. 32, 177–189. doi: 10.5093/pi2023a12 37691715 PMC10484026

[B30] Spaas C. Verbiest S. Smet d. e. Kevers S. Missotten R. L. De Haene L. (2022). Working with the encounter: a descriptive account and case analysis of school-based collaborative mental health care for refugee children in Leuven, Belgium. Front. Psychol. 13:806473. doi: 10.3389/fpsyg.2022.806473 35356344 PMC8959124

[B31] Thoma N. Langer P. (2022). Educational transitions in war and refugee contexts: youth Biographies in Afghanistan and Austria. Soc. Incl. 10, 302–312. doi: 10.17645/si.v10i2.5156

[B32] UN (2019). Refugee education in crisis: More than half of the world's school-age refugee children do not get an education. New York, NY: United Nations. Available online at: https://www.un.org/en/academic.impact/refugee-education-crisis-more-half-worlds-school-age-refugee-children-do-not-get-education (Accessed June 5, 2024).

[B33] UN Refugee Agency UNHCR (2026). Learning for a future: refugee education in developing countries. https://www.unhcr.org/sites/default/files/legacy-pdf/4a1d5ba36.pdf (Accessed March 08, 2026).

[B34] UNESCO (2026). Beyond access: Rethinking refugee education. Available online at: https://www.unesco.org/sdg4education2030/eu/articles/beyond-access-rethining-refugee-education (Accessed July 9, 2026).

[B35] UNHCR (2017). Left behind. New York, NY: United Nations. Available online at: https://www.unhcr.org/left-behind/ (Accessed July 6, 2026).

[B36] UNHCR (2020). Why education for refugee children matters. Geneva: United Nations High Commission for Refugees. Available online at: https://www.unhcr.org/starting-out-why-education-refugee-children-matters (Accessed March 28, 2024).

[B37] UNHCR (2023). Build better futures: education. Geneva: United Nations High Commission for Refugees. Available online at: https://www.unhcr.org/what-we-do/build-better-futures/education (Accessed March 30, 2024).

[B38] UNHCR (2024). About migration and human rights. Geneva: United Nations High Commission for Refugees. Available online at: https://www.unhcr.org/herturn (Accessed April 27, 2024).

[B39] UNICEF (1989). The UN Convention on the Rights of the Child. Rules for the protection of children worldwide. New York, NY: United Nations Children's Fund. Available online at: https://www.unicef.de/informieren/aktuelles/presse/-/gefluechtete-kinder-in-aufnahmeeinrichtungen-einschraenkungen-beim-zugang-zu-bildung-/386484 (Accessed March 31, 2024).

